# ITGAX promotes gastric cancer progression via epithelial-mesenchymal transition pathway

**DOI:** 10.3389/fphar.2024.1536478

**Published:** 2025-01-08

**Authors:** Jiali Hu, Jing Cao, Shanshan Huang, Yang Chen

**Affiliations:** ^1^ Department of Oncology, The Second Affiliated Hospital of Nanchang University, Nanchang, China; ^2^ School of Pharmacy, Jiangxi University of Traditional Chinese Medicine, Nanchang, China; ^3^ Department of Oncology, The First Affiliated Hospital of Nanchang University, Nanchang, China; ^4^ Ganjiang Chinese Medicine Innovation Center, Nanchang, China; ^5^ Key Laboratory of Phytochemistry and Natural Medicines, Dalian Institute of Chemical Physics, Chinese Academy of Sciences, Dalian, China

**Keywords:** ITGAX, gastric cancer, EMT, cell migration, tumorigenesis

## Abstract

Gastric cancer is the fifth most common cancer and the fourth leading cause of cancer-related deaths worldwide, accounting for nearly 800,000 fatalities annually. ITGAX (Integrin alpha X) is closely associated with immune cells, such as macrophages and dendritic cells. Its involvement in gastric cancer was identified through an analysis of The Gene Expression Omnibus (GEO) database, which highlighted *ITGAX* as one of four key gastric cancer-related genes. Our study demonstrates that ITGAX expression is significantly elevated in tumor tissues compared to normal tissues and is positively correlated with clinical prognosis in gastric cancer patients from the GEO database. Moreover, ITGAX enhanced cell proliferation, invasion, and tumorigenic capacity in mouse models. Furthermore, we explored the underlying role of ITGAX using Kyoto Encyclopedia of Genes and Genomes (KEGG) and protein-protein interaction networks (PPI) analysis. Our findings reveal that ITGAX promotes gastric cancer progression by driving epithelial-mesenchymal transition pathway (EMT), suggesting its potential as a biomarker for early diagnosis and prognosis in gastric cancer.

## 1 Introduction

Gastric cancer remains a major global health challenge, ranking as the fifth most common malignancy and the fourth leading cause of cancer-related deaths worldwide, accounting for approximately 800,000 deaths annually (Yang et al., 2023). While significant progress has been made in early detection and treatment, the prognosis for gastric cancer remains poor, with a 5-year survival rate below 30% (Smyth et al., 2020). Late-stage diagnoses, tumor recurrence, and metastasis are the primary reasons for poor outcomes (Plummer et al., 2015). Despite the overall declining incidence in recent decades, largely attributed to reduced *Helicobacter pylori* infection, improved food storage technologies, and dietary awareness, this decline is predominantly observed in distal (non-cardia) gastric cancer (Correa, 2013). In contrast, the incidence of proximal (cardia) gastric cancer has shown a upward trend, closely associated with the rising obesity and gastroesophageal reflux disease (GERD), both of which contribute to chronic inflammation and tumorigenesis in the gastric cardia (Lagergren et al., 2017).

Importantly, the incidence of gastric cancer increased among younger populations, particularly women under the age of 50, which diverges from traditional patterns predominantly affecting older males (Anderson et al., 2018). This emerging trend raises concerns about alternative etiological factors, including autoimmune gastritis, genetic susceptibility, and shifts in the gastric microbiome (Noto and Peek, 2017). Chronic inflammation, driven by *H. pylori* infection or environmental triggers, may play a critical role by promoting epithelial cell transformation and tumor progression (Cullin et al., 2021). These epidemiological and demographic shifts underscore the need for deeper insights into the molecular mechanisms driving gastric cancer progression to improve early detection and therapeutic strategies.

Recent advances in bioinformatics and high-throughput sequencing have enabled the identification of novel biomarkers and therapeutic targets in gastric cancer. Co-expression network analysis of datasets from the GEO database has identified four key genes involved in gastric cancer progression including *ITGAX*, chemokine (C-C motif) ligand 14 (*CCL14*), alcohol dehydrogenase iron-containing protein 1 (*ADHFE1*), and Homeobox B13 (*HOXB13*) (Rezaei et al., 2022). Among these, ITGAX has emerged as a significant candidate (He et al., 2019; Zhang and Horvath, 2005). ITGAX is a transmembrane glycoprotein belonging to the integrin family, is widely recognized as a marker for immune cells, including macrophages and dendritic cells (Hynes, 2002). Integrins are critical for cell adhesion, migration, and immune surveillance, but their aberrant expression in cancer can promote tumor growth, angiogenesis, and metastasis (Desgrosellier and Cheresh, 2010).

Although ITGAX is well-studied in inflammatory conditions, such as neuroinflammation, non-alcoholic steatohepatitis, and dermatitis-like inflammation (Kalluri and Weinberg, 2009; Yan et al., 2022; Ullah et al., 2024), its functional significance in gastric cancer remains poorly understood. ITGAX may play a critical role in cancer progression by modulating the EMT pathway. EMT is a key process in tumorigenesis that drives cancer cell invasion, metastasis, and chemoresistance. During EMT, epithelial cells lose adhesion properties and acquire mesenchymal traits, such as increased motility and invasiveness (Lamouille et al., 2014). EMT is regulated by transcription factors like Snail, Slug, and Twist, which are often activated by oncogenic pathways, including Wnt/β-catenin, PI3K/AKT, and TGF-β signaling (Puisieux et al., 2014; Zhou et al., 2017). Moreover, EMT promotes cancer stem cell-like properties, contributing to tumor recurrence and therapy resistance (Mani et al., 2008).

Our study demonstrates that ITGAX expression is significantly elevated in gastric cancer tissues compared to normal tissues and correlates with poor clinical prognosis. Functional assays reveal that ITGAX overexpression enhances cell proliferation, migration, invasion, and tumorigenic capacity using *in vitro* and *in vivo* models, while ITGAX silencing reverses these effects. Mechanistically, ITGAX regulates the EMT pathway, as evidenced by reduced E-cadherin expression and increased N-cadherin and vimentin levels upon ITGAX overexpression, consistent with an EMT-promoting phenotype. These findings highlight ITGAX as a key driver of gastric cancer progression, facilitating EMT and enhancing tumor invasion and metastasis. Additionally, its strong association with immune regulation suggests a dual role in modulating the tumor microenvironment. Further investigation into the ITGAX-EMT axis could provide novel insights into the molecular mechanisms underlying gastric cancer and identify promising therapeutic targets. In conclusion, ITGAX holds significant potential as a biomarker for early diagnosis, prognosis, and treatment in gastric cancer, particularly for aggressive or metastatic cases.

## 2 Materials and methods

### 2.1 Data collection and analysis

We collected data from the GSE122401 dataset available on the NCBI website (https://www.ncbi.nlm.nih.gov/) for our analysis. Box plots were generated, and enrichment analysis was performed using R version 2.1, utilizing the KEGG database.

### 2.2 Cell culture

Human gastric mucosal epithelial cell GES-1 and the human gastric cancer cell lines LMSU, ECC10, HS766.T, and SH-10-TC were obtained from the ATCC cell bank (https://www.atcc.org/). The cells were cultured in RPMI 1640 medium supplemented with 15% fetal bovine serum and 1% penicillin/streptomycin (P/S) at 37°C in a 5% CO_2_ incubator.

### 2.3 Stable cell line

The *ITGAX* gene was synthesized by Beijing Tsingke Biotech Co., Ltd. (https://www.tsingke.com.cn/). After preparing DNA mixture, the cells were incubated at room temperature for 20 min and then cultured in a CO_2_ incubator at 37°C. Twenty hours post-transfection, the cell culture medium was replaced with fresh medium. After 48 h, the supernatant was collected, centrifuged, and filtered at 4°C. The virus was aliquoted into 50 μL per tube for subsequent experiments. LMSU and SH-10-TC cells were inoculated into 24-well plates and cultured overnight. They were then infected with lentivirus. Twenty-4 hours post-infection, the medium was replaced with complete culture medium, and the cells were incubated at 37°C. After 48 h, complete culture medium containing puromycin was added to obtain stable cell lines.

### 2.4 Real-time quantitative PCR (qRT-PCR)

RNA was extracted using the TRIzol method. Reverse transcription of mRNA was carried out using the PrimeScript™ RT Reagent Kit (Takara, Kusatsu, Japan) with the gDNA Eraser (Perfect Real Time) method. Subsequently, qPCR was conducted using the obtained cDNA. The experimental procedure followed the protocol outlined in the TB Green^®^ Premix Ex Taq™ II (Tli RNase H Plus) kit. The primers were listed as *ITGAX*-F: CCT​TGG​TCC​AGC​TCT​TCC​TG, *ITGAX*-R: CCC​AGG​AGT​TGG​CAT​ACT​GG.

### 2.5 Western blotting

Total protein was extracted from the cells and centrifuged at 4°C. The supernatant was loaded onto a 10% SDS-PAGE gel and subjected to protein electrophoresis. Following electrophoresis, proteins were transferred to a membrane, then the membrane was blocked with 5% normal calf serum for 1 h and washed three times with Tris-buffered saline with Tween 20 (TBST). Next, the membrane was incubated with the primary antibody at 4°C overnight. After washing with TBST, the membranes were incubated with secondary antibodies at room temperature for 2 h. Enhanced chemiluminescence (ECL) was used for detection, and gray values were measured using an automatic gel imaging analyzer. The following antibodies were used in this experiment: GAPDH (Proteintech, 60004-1-Ig), E-cadherin (Proteintech, 60335-1-Ig), N-cadherin (Proteintech, 66219-1-Ig), Vimentin (Proteintech, 60330-1-Ig), ITGAX (Proteintech, 60258-1-Ig).

### 2.6 Cell viability assay

Cell viability was assessed using the Cell Counting Kit-8 (CCK-8) assay. Cell suspensions were seeded in 96-well plates and cultured at 37°C in a 5% CO_2_ incubator. After the designated incubation period, 10 µL of CCK-8 solution was added to each well. Following a 2-hour incubation, the absorbance was measured at 450 nm.

### 2.7 Flow cytometry assay

Apoptosis detection was performed using the TransDetect^®^ Annexin V-FITC/PI Cell Apoptosis Detection Kit (Transgen, Beijing, China). The cells were stained according to the protocol, and the fluorescence signals were detected using flow cytometry.

### 2.8 Transwell assay

In the transwell assay, 4 × 10⁴ cells in 200 µL of medium without fetal bovine serum (FBS) were added to the upper chamber, and 800 µL of medium containing 10% FBS was added to the lower chamber. For the invasion experiment, matrix glue was added to the medium, whereas no matrix glue was added for the migration experiment. The cells were then cultured in a 37°C incubator to facilitate matrix penetration. After 48 h, the cells in the upper chamber were fixed with 4% paraformaldehyde and stained with 0.5% crystal violet solution. Excess fluid was removed from the chambers, the upper chamber was washed with 300 µL PBS, and the lower chamber was washed with 700 µL PBS. The stain was removed using 40% acetic acid. Non-migrated cells on the upper chamber surface were then removed, and migrated cells on the lower surface were visualized and captured using an optical microscope. Six random fields of view (20 × magnification) were examined.

### 2.9 Colony formation assay

The cell suspension was inoculated with a gradient dilution and cultured in a cell incubator at 37°C with 5% CO_2_ and saturated humidity for 2 weeks. The culture was stopped once visible colonies appeared on the dish. The supernatant was discarded, and the cells were carefully rinsed with PBS. Methanol was added for fixation for 15 min, followed by the addition of an appropriate amount of Giemsa dye solution for 30 min. The dye was then gently washed off with water, and the cells were photographed.

### 2.10 EdU assay

Cell proliferation was detected using an EdU Cell Proliferation Detection Kit (Shanghai Shenggong Company, Shanghai, China). Adherent cells were incubated with EdU solution for 2 h. After removing the EdU solution, the cells were fixed with formaldehyde and treated with glycine and Triton X-100. Following fixation, EdU was added, and the cells were incubated for 30 min under light-avoiding conditions. After washing, the cells were incubated with Hoechst 33,342 for another 30 min under light-avoiding conditions. Finally, the cells were washed again and photographed using a fluorescence microscope with excitation wavelengths of 541 nm and 350 nm, and emission wavelengths of 567 nm and 461 nm.

### 2.11 Animal experiment

Specific-pathogen-free (SPF) female nude mice (BALB/C-nu), aged 4–6 weeks and weighing approximately 20 g, were purchased from GemPharmatech Co., Ltd. (SCXK(SU)2023-0009). Upon arrival, the animals were housed in the Laboratory Animal Research Center of The First Affiliated Hospital of Nanchang University (SYXK(GAN)2021-0003) and acclimated to the environment under appropriate feeding conditions for 1 week. All animal experiments were approved by the Institutional Animal Care and Use Committee of The First Affiliated Hospital of Nanchang University (Approval number: CDYFY-IACUC-202407QR008).

The mice were randomly assigned to groups for subcutaneous tumor induction and maintained at 25°C with 40%–60% humidity and a 12-h light/dark cycle. Tumor cells were injected subcutaneously into the mice. Each treatment group consisted of three mice. The body weight and tumor volume of the mice were measured at 0, 3, 7, 14, 21, and 28 days post-injection. The long and short diameters of the tumors were measured using Vernier calipers, and tumor volume was calculated with the formula: V = π/6 × L (long diameter) × W (short diameter) × H (height). After 28 days, the mice were euthanized, and the tumors were excised, photographed, and weighed to determine the mass of the subcutaneous tumor tissue.

### 2.12 HE staining

Tissue sections were stained with hematoxylin, rinsed with water for 1 min to remove excess dye, and differentiated with 1% hydrochloric acid. The sections were then stained with eosin for 1 min, rinsed with water, sealed with neutral gum, and observed under a microscope before being photographed.

### 2.13 Statistical analysis

Statistical analysis was performed using GraphPad Prism software (version 8.0.2, GraphPad Software, USA). The mean and standard deviation (SD) were calculated for the data. Statistical differences between experimental groups were analyzed using Student’s t-test. All data are presented as mean ± SD, with statistical significance defined as *p* < 0.05.

## 3 Results

### 3.1 ITGAX is highly expressed in gastric cancer

**FIGURE 1:**
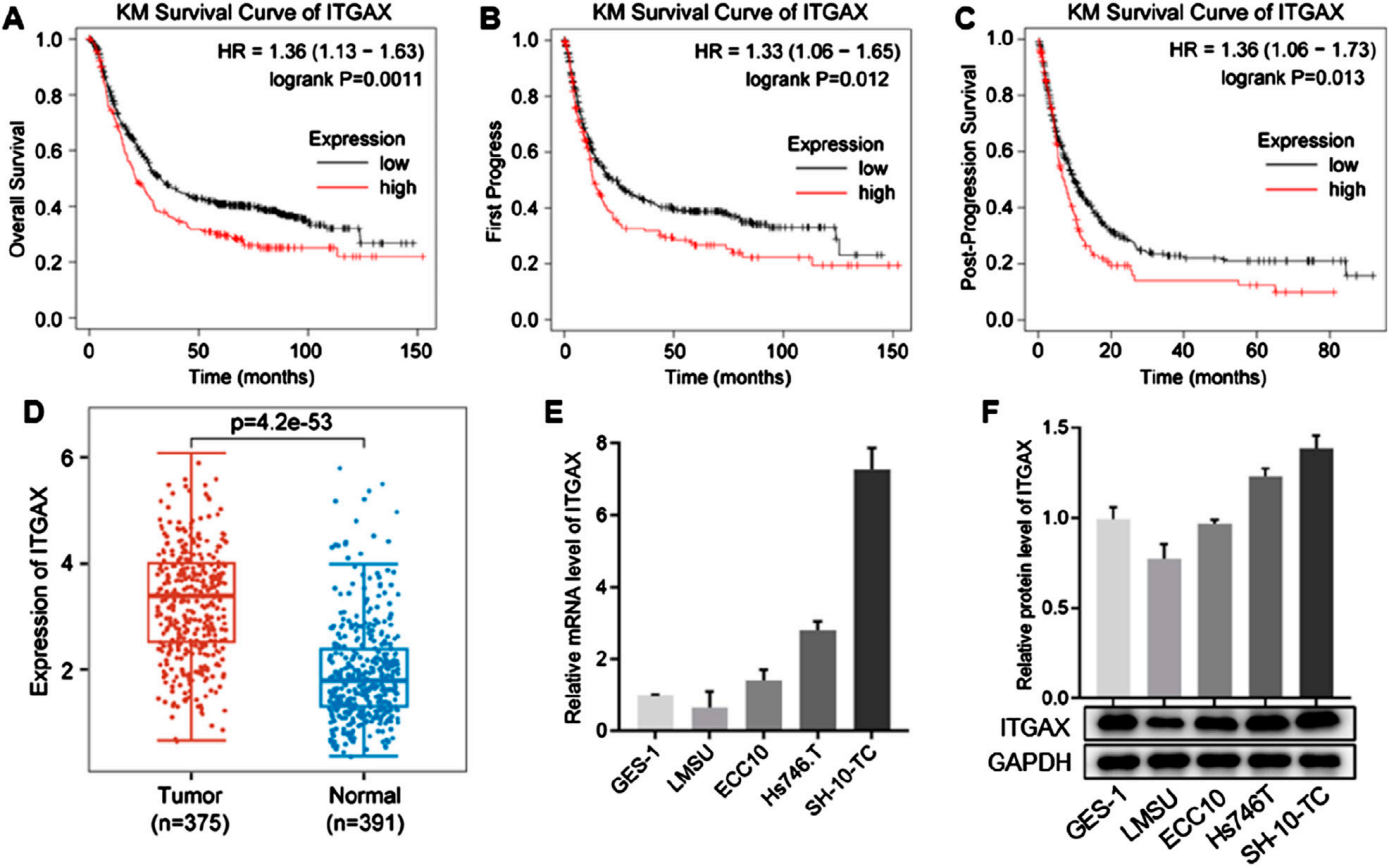
ITGAX is positively correlated with gastric cancer. **(A–C)** *Kaplan-Meier* plots showing the association between ITGAX expression and survival analysis in patients with gastric cancer. **(D)** Box plot of ITGAX expression in peritumoral and gastric cancer tissues. **(E, F)** mRNA and protein expression levels of ITGAX in gastric cancer cell lines.

We downloaded the GSE122401 dataset from NCBI for expression and survival analysis. The results indicated that high ITGAX expression was associated with a poor survival rate in patients with gastric cancer (Figures 1A–C). Additionally, ITGAX expression was significantly higher in patients with gastric cancer compared to the healthy group (Figure 1D). We analyzed ITGAX expression levels in the human gastric mucosal epithelial cell line (GES-1) and various human gastric cancer cell lines (LMSU, ECC10, HS746.T, and SH-10-TC). Notably, ITGAX expression was significantly lower in LMSU cells compared to GES-1 cells, while it was significantly higher in HS746.T and SH-10-TC cells, both at the mRNA and protein levels (Figures 1E, F). Among the four cancer cell lines, ITGAX exhibited the highest expression level in SH-10-TC cells and the lowest in LMSU cells.

### 3.2 ITGAX overexpression increases gastric cancer cell migration and invasion

**FIGURE 2:**
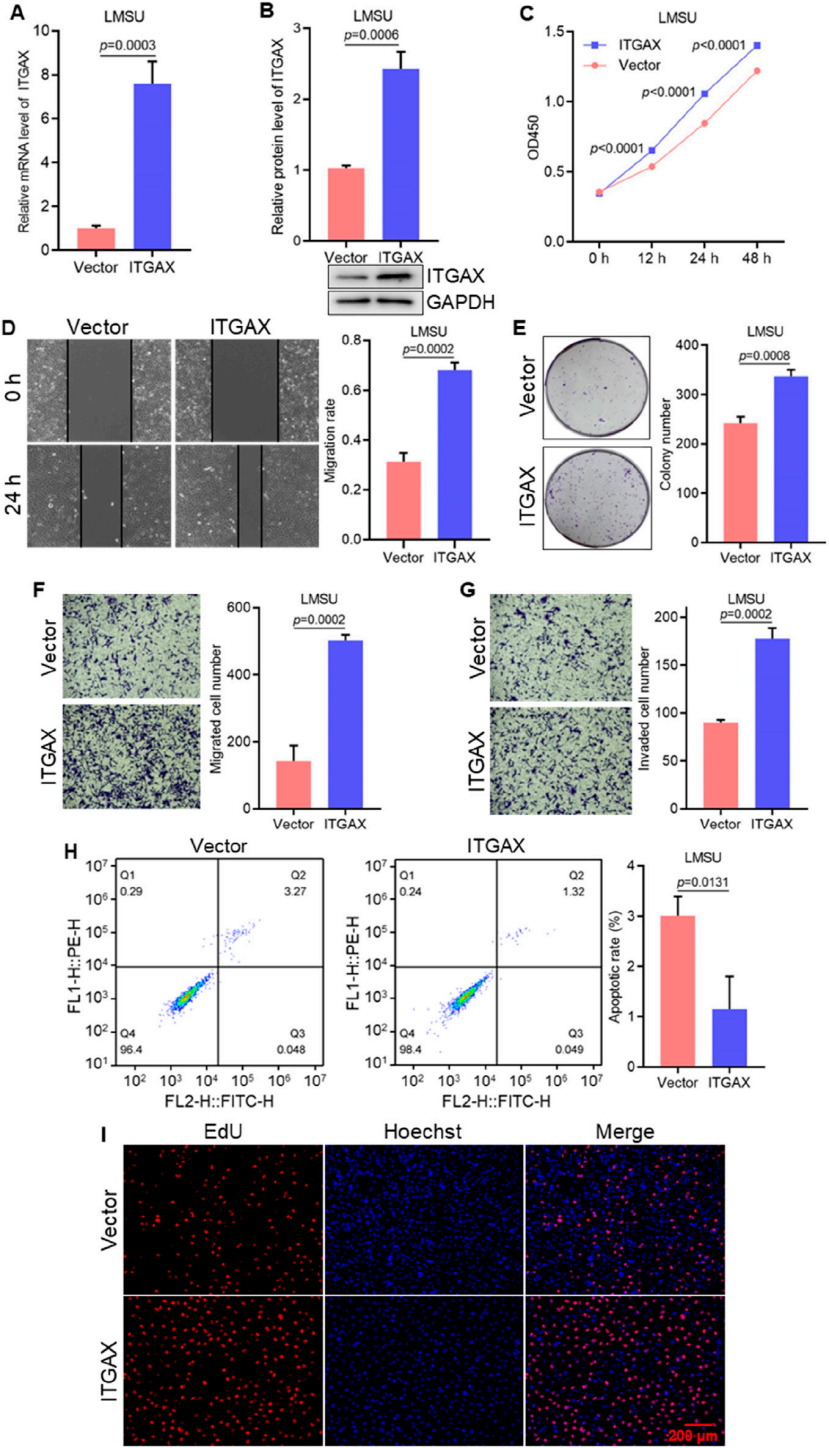
ITGAX overexpression promotes cell growth and migration in gastric cancer cells. **(A, B)** mRNA and protein levels in ITGAX-overexpressed LMSU cells, assessed by qRT-PCR and Western blotting assays. **(C)** Cell viability of ITGAX-overexpressed LMSU cells measured by CCK-8 assay. **(D)** Colony formation assay evaluating the proliferation ability of ITGAX overexpressed LMSU cells. **(E, F)** Cell migration ability of ITGAX-overexpressed LMSU cells assessed using the scratch assay and Transwell assay. **(G)** Cell invasion ability of ITGAX-overexpressed LMSU cells measured by the Transwell assay. **(H)** Apoptosis rate of ITGAX-overexpressed LMSU cells detected by flow cytometry. **(I)** EdU staining used to assess proliferation in ITGAX-overexpressed LMSU cells.

Stable ITGAX-overexpressing LMSU cells were established, and the mRNA and protein levels of ITGAX were confirmed (Figures 2A, B). We then investigated the cellular functions of ITGAX overexpression in LMSU cells using various cellular assays. In the cell viability assay, the OD450 value in ITGAX-overexpressing LMSU cells was significantly higher than that of control LMSU cells (Figure 2C). Colony formation assays showed a significant increase in the colony numbers in ITGAX-overexpressing LMSU cells compared to the control LMSU cells (Figure 2D). In scratch and transwell assays, high ITGAX expression correlated with increased invasion ability (Figures 2E, F). The transwell assay also revealed that ITGAX overexpression promoted cell migration (Figure 2G). Apoptosis detection indicated that the apoptosis rate of control LMSU cells was 3.34%, whereas it was significantly lower in ITGAX-overexpressing LMSU cells at 0.76% (Figure 2H). The EdU assay was employed to assess cell proliferation. Hoechst staining showed no significant difference in the number of cells, while EdU staining revealed a substantial difference in proliferating cells, with a significantly higher number of proliferating ITGAX-overexpressing LMSU cells compared to control LMSU cells (Figure 2I).

### 3.3 Knockdown of ITGAX hinders cell growth and migration in gastric cancer

**FIGURE 3:**
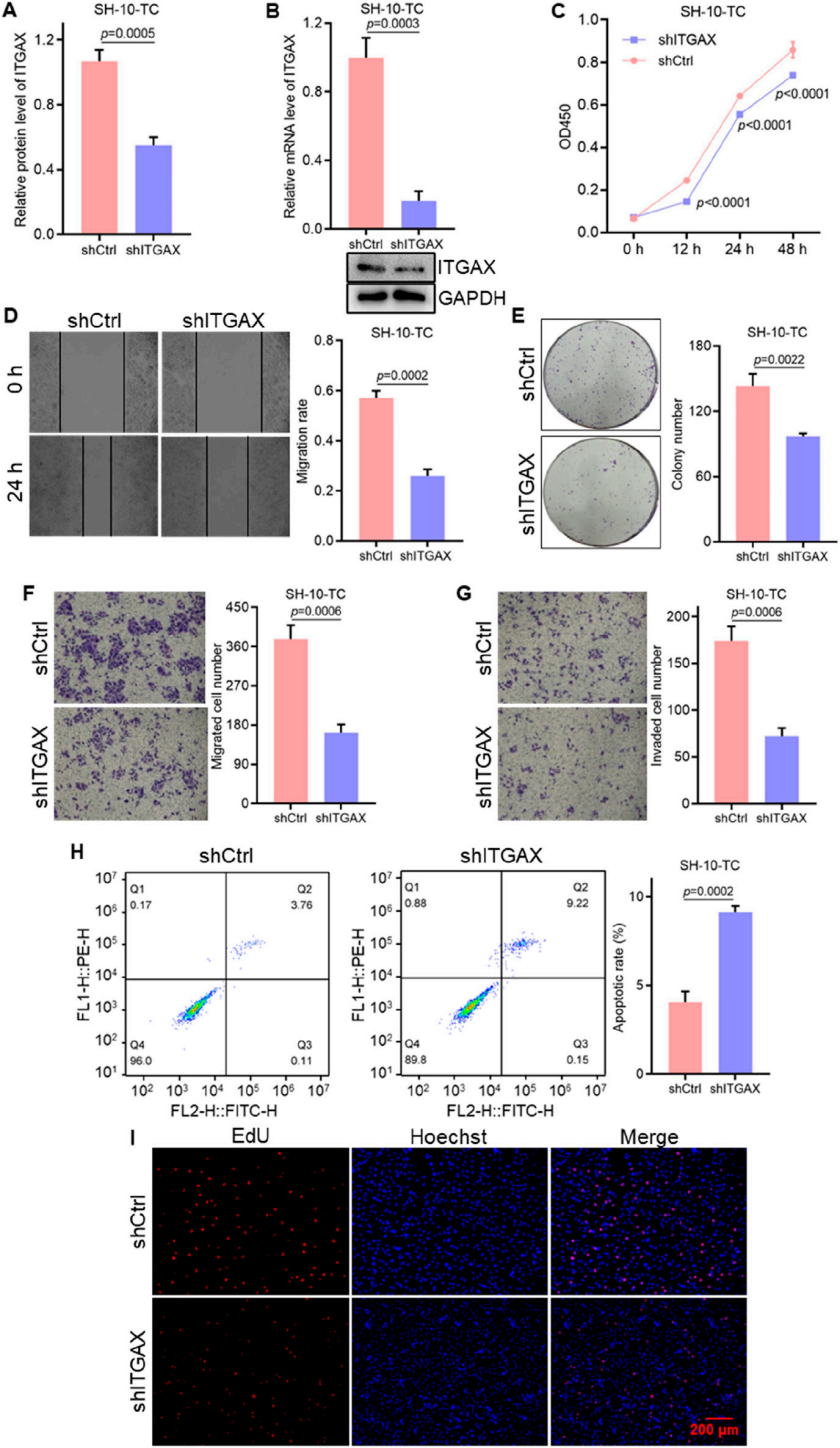
The knockdown of ITGAX hinders gastric cancer cell growth and migration. **(A, B)** mRNA and protein levels in ITGAX knockdown SH-10-TC cells, assessed by qRT-PCR and Western blotting assays. **(C)** Cell viability of ITGAX knockdown SH-10-TC cells measured by CCK-8 assay. **(D)** Colony formation assay evaluating the proliferation ability of ITGAX knockdown SH-10-TC cells. **(E, F)** Cell migration ability of ITGAX knockdown SH-10-TC cells assessed using the scratch assay and Transwell assay. **(G)** Cell invasion ability of ITGAX knockdown SH-10-TC cells measured by the Transwell assay. **(H)** Apoptosis rate of ITGAX knockdown SH-10-TC cells detected by flow cytometry. **(I)** EdU staining used to assess proliferation in ITGAX knockdown SH-10-TC cells.

Stable ITGAX knockdown SH-10-TC cells were established and the mRNA and protein levels of ITGAX were detected (Figures 3A, B). The effects of ITGAX knockdown in SH-10-TC cells were then evaluated using various cellular assays. Cell viability was significantly decreased in the ITGAX knockdown SH-10-TC group (Figure 3C). Colony formation assays showed a decrease in colony numbers in the ITGAX knockdown SH-10-TC group (Figure 3D). Similarly, cell migration ability showed a significant decline in the ITGAX knockdown SH-10-TC group (Figures 3E, F), and cell invasion ability was decreased with ITGAX knockdown (Figure 3G). Apoptosis detection assays revealed that the apoptosis rate of control SH-10-TC cells was 2.74%, whereas it was significantly higher at 9.08% in ITGAX knockdown SH-10-TC cells (Figure 3H). Hoechst staining showed no significant difference in the number of cells in the visual field. However, EdU staining demonstrated a significant reduction in the number of proliferating cells in the ITGAX knockdown SH-10-TC group (Figure 3I), Therefore, the downregulation of ITGAX expression resulted in decreased cell viability, colony formation, invasion, proliferation, and migration abilities in gastric cancer cells.

### 3.4 ITGAX plays an important role in EMT in gastric cancer

**FIGURE 4:**
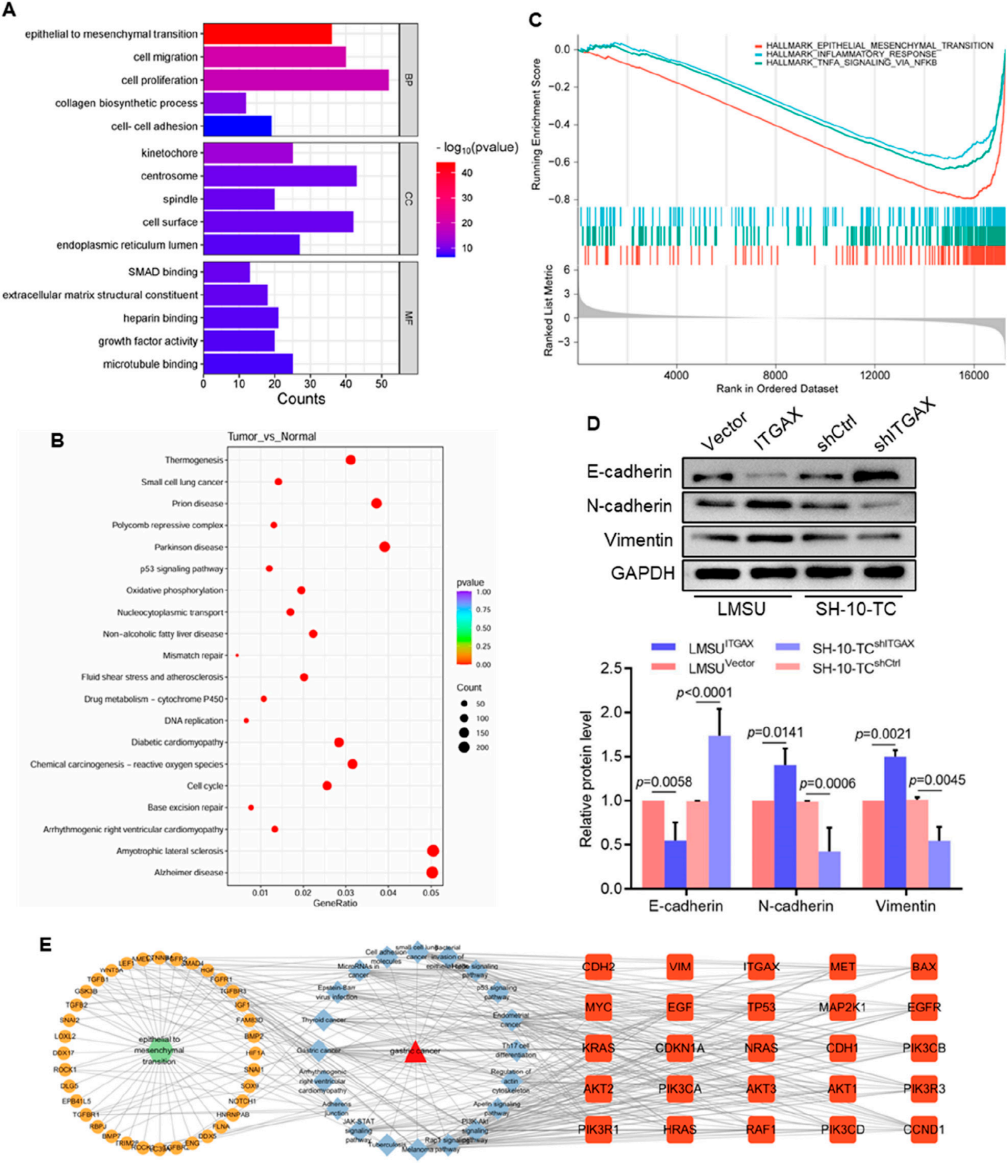
Correlation analysis of ITGAX and the epithelial–mesenchymal transition pathway in gastric cancer. **(A)** Gene Ontology (GO) analysis of pathway enrichment correlation. **(B)** KEGG pathway enrichment analysis. **(C)** The functional enrichment analysis using gene set enrichment analysis (GSEA). **(D)** Protein expression levels of E-cadherin, N-cadherin, and vimentin detected using Western blotting. **(E)** Protein-protein interaction (PPI) network diagram of multi-omics analysis.

Enrichment analysis of the dataset revealed that, compared with the healthy group, data from patients with gastric cancer were primarily associated with genes involved in reactive oxygen species production and the cell cycle (Figures 4A–C), which revealed significant differences in gene expression between the high and low ITGAX expression groups, particularly in key biological processes such as EMT, inflammatory response, and NF-κB signaling, potentially contributing to tumor progression and immune modulation. Given the effects of ITGAX expression on cell activity, apoptosis, invasion, colony formation, and proliferation, ITGAX might influence cellular function through the EMT pathway. To investigate this, we examined the expression levels of E-cadherin, N-cadherin, and vimentin in ITGAX-overexpressing LMSU cells and ITGAX knockdown SH-10-TC cells. The results indicated that E-cadherin expression was significantly decreased in ITGAX-overexpressing LMSU cells compared to the control LMSU cells, while it was elevated in ITGAX knockdown SH-10-TC cells compared to the control SH-10-TC cells. In contrast, the expressions of N-cadherin and vimentin were significantly higher in ITGAX-overexpressing LMSU cells than in the control LMSU cells, and lower in ITGAX knockdown SH-10-TC cells than in the control SH-10-TC cells (Figure 4D). Furthermore, our findings demonstrated that ITGAX regulates epithelial-mesenchymal transition (EMT) and immune modulation, which mediate tumor progression in gastric cancer. Potential targets of ITGAX were identified through protein-protein interaction (PPI) network analysis, providing further insights into its functional roles (Figure 4E). Overall, ITGAX overexpression led to decreased E-cadherin expression and increased N-cadherin and vimentin expression, suggesting a promotion of the EMT process. Conversely, ITGAX knockdown resulted in higher E-cadherin expression and lower N-cadherin and vimentin expression, indicating an inhibition of the EMT pathway.

### 3.5 ITGAX advances tumor progression of gastric cancer *in vivo*

**FIGURE 5:**
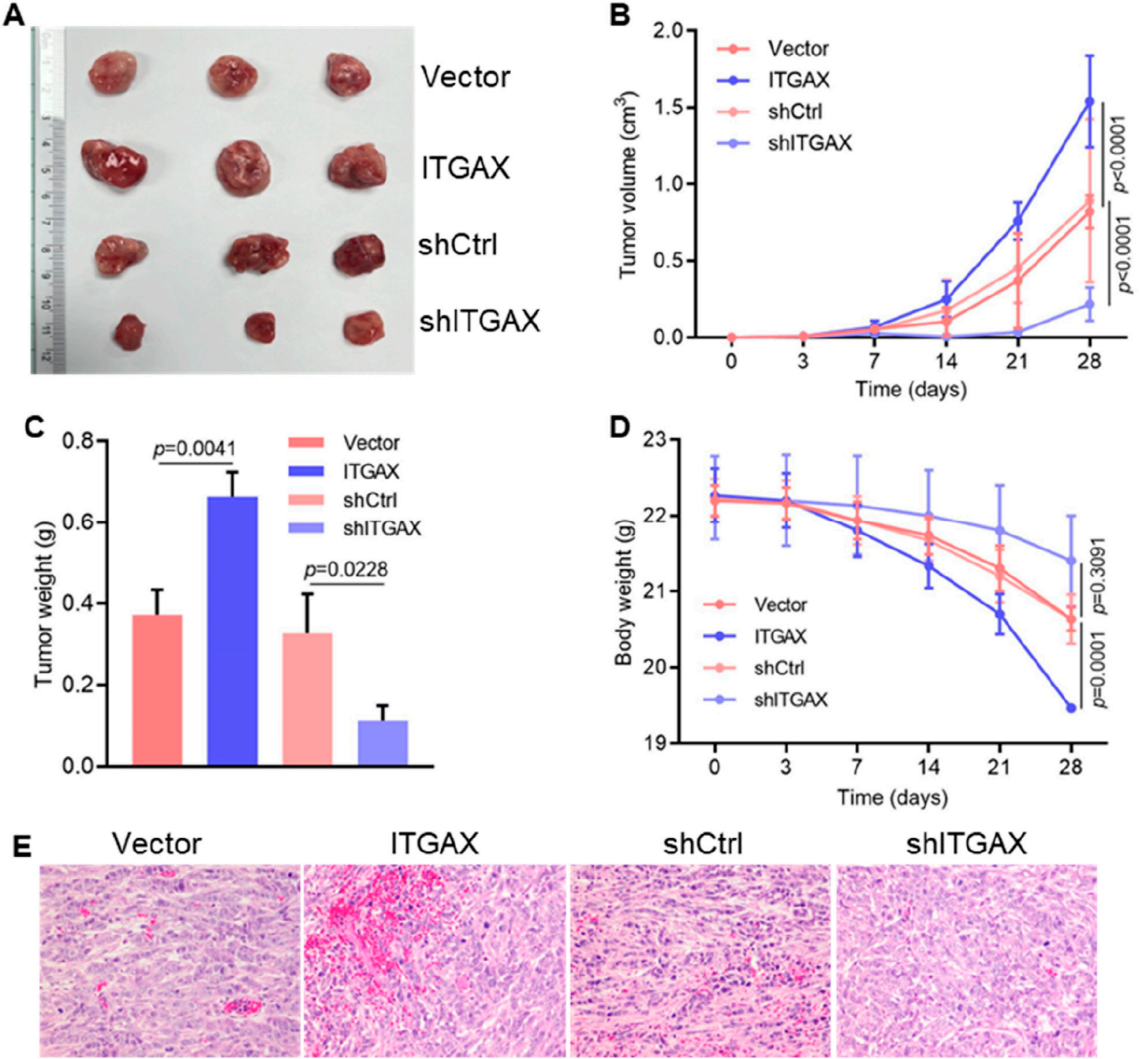
ITGAX advances tumor progression of gastric cancer in mice. **(A)** Representative photographs of primary tumors injected with ITGAX overexpressed or knockdown gastric cells in mice. **(B)** Measurement of tumor volume in mice. **(C)** Effect of ITGAX on tumor weight in mice. **(D)** Body weight changes of mice after subcutaneous injection of ITGAX-overexpressed or knockdown gastric cells. **(E)** Pathological analysis of tumor tissues using H&E staining.

To evaluate the tumorigenic ability of ITGAX in gastric cancer cells, 4 cell lines, including ITGAX-overexpressing LMSU cells and ITGAX knockdown SH-10-TC cells along with their respective control cells, were subcutaneously injected into nude mice. Changes in tumor volume, tumor weight, and body weight were monitored, and tumor tissue was analyzed. Notably, the tumor volume in mice increased gradually over time. Mice with upregulated ITGAX expression exhibited the fastest tumor growth, while the knockdown group showed the slowest growth (Figures 5A, B). Tumor weight followed the same increasing trend as tumor volume (Figure 5C). Conversely, the body weight of mice showed an opposite trend, decreasing over time (Figure 5D). HE staining revealed notable changes in vascular structures within the tumors of the ITGAX overexpression and knockdown groups compared to their respective controls. In the ITGAX overexpression group, vessel diameters significantly expanded. Conversely, in the ITGAX knockdown group, vascular structures remained largely unchanged, and the number of blood cells continued to be low (Figure 5E).

## 4 Discussion

**FIGURE 6:**
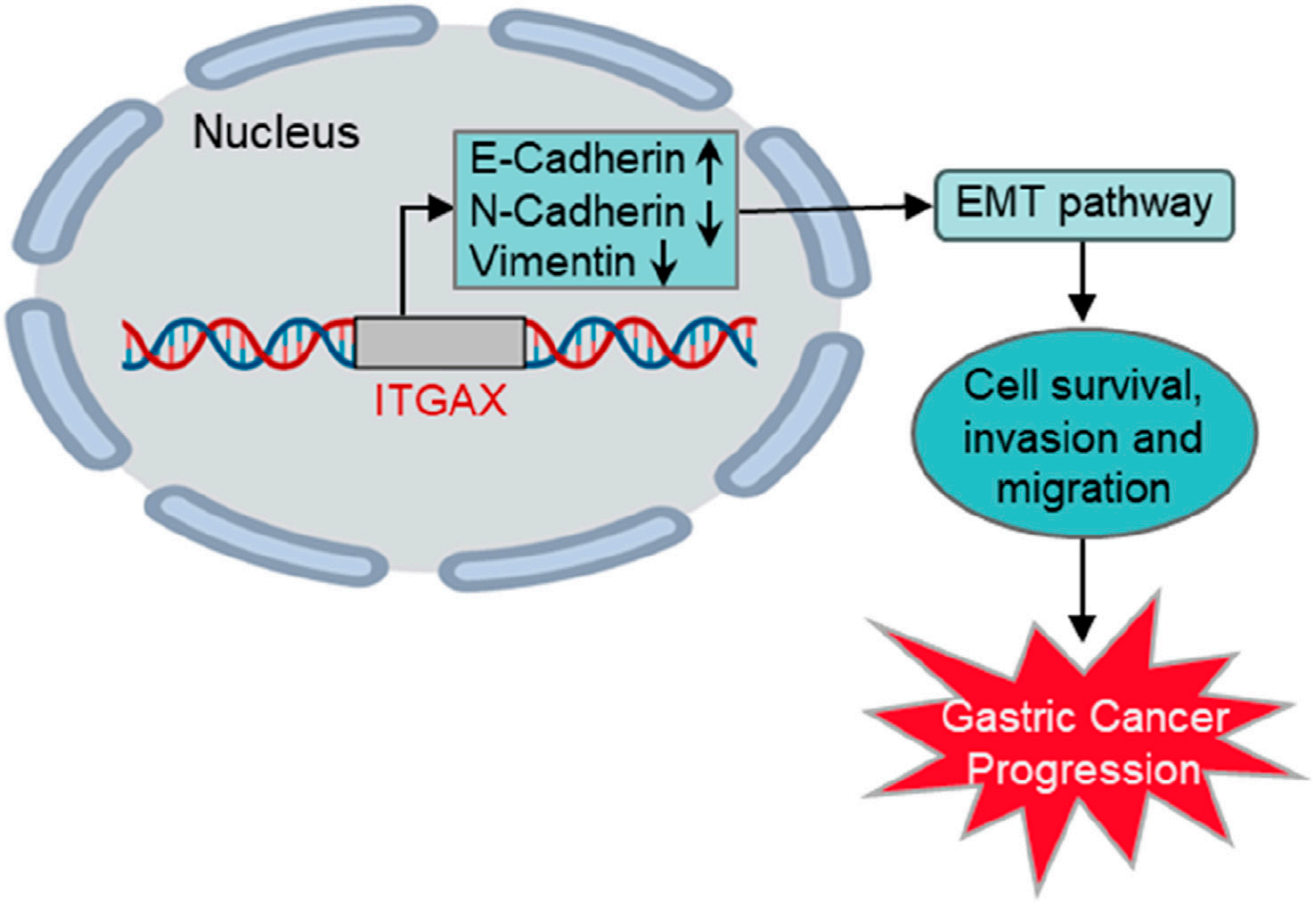
Schematic illustration of ITGAX-enhanced gastric cancer progression via the EMT pathway.

ITGAX, a member of the integrin family, is a transmembrane glycoprotein that functions as an extracellular matrix receptor and plays a critical role in cell adhesion, migration, and immune response. Aberrant expression of integrins is frequently associated with tumor progression, angiogenesis, and metastasis. In this study, we demonstrated that ITGAX overexpression in gastric cancer cells significantly enhanced proliferation, migration and invasion while reducing apoptosis, highlighting its oncogenic potential. Conversely, ITGAX knockout inhibited these processes, further supporting its functional relevance in gastric cancer progression (Figure 6).

ITGAX has previously been implicated in multiple cancers and inflammatory diseases. For example, ITGAX is recognized as a novel susceptibility gene for prostate cancer, where it contributes to tumor aggressiveness and angiogenesis (Wang et al., 2019; Williams et al., 2014). Similarly, it enhances tumor growth and angiogenesis in ovarian cancer, emphasizing its role in facilitating vascular development, a critical process for tumor survival and expansion (Zhang et al., 2019). Beyond cancer, ITGAX plays a significant role in atherosclerosis and ischemic stroke through dendritic cell regulation and promotion of atherosclerotic tissue formation (Wang et al., 2023; Cai et al., 2023). ITGAX and CCR1 were identified as potential biomarkers for atherosclerosis (Yan et al., 2022), while macrophage-related genes, including ITGAX/CD11c and interferon regulatory factor 5, are strongly associated with symptomatic carotid artery disease (Edsfeldt et al., 2022). ITGAX also modulates immune pathways by activating immune-associated B cells, programmed cell death protein 1 (PDCD1) peripheral T helper cells, and follicular T helper cells (Zhang et al., 2019). Additionally, ITGAX plays a role in inflammatory pathways in chronic kidney disease, ulcerative colitis (Liang et al., 2023), and neurodegenerative diseases like Alzheimer’s disease and cerebral amyloid angiopathy, where its overexpression in microglia contributes to neuroinflammation (Lopes et al., 2024). These findings suggest that ITGAX functions as both an immune regulator and a driver of angiogenesis across diverse pathological conditions. Despite its well-documented involvement in immune regulation, the specific mechanisms linking ITGAX to cancer progression, particularly in gastric cancer, remain poorly understood.

Given its oncogenic role, we investigated the relationship between ITGAX and the EMT pathway. EMT is a critical process in cancer progression that enables epithelial cells to acquire mesenchymal traits, enhancing migration, invasion, and resistance to apoptosis. Our findings indicate that ITGAX facilitates gastric cancer cell invasion and proliferation by modulating the EMT signaling pathway. While ITGAX involvement in EMT has not been previously reported, its association with angiogenesis through VEGF expression and the PI3K/Akt pathway is well established (Zhang et al., 2019). Additionally, ITGAX is implicated in inflammatory signaling pathways, such as TNF-α (Wang et al., 2023) and AMPK-ULK1 autophagy axis (Zhang et al., 2022). EMT activation is also associated with various cellular processes, including post-transcriptional modification (Gao et al., 2023; Liu et al., 2023), cytoskeletal rearrangement (He et al., 2014; Hu J. L. et al., 2015; Chen et al., 2017), extracellular matrix remodeling (Polyak and Weinberg, 2009), RNA processing (Zhang et al., 2021; Hu et al., 2020), and drug treatment (Chen et al., 2024; Dai et al., 2024; Zhang et al., 2024). Autophagy, is a key EMT regulator (Que et al., 2022), is influenced by E-cadherin downregulation, which has been associated with lymphatic proliferation and poor prognosis in malignant tumors (Russo et al., 2024; Schipper et al., 1991; Takayama et al., 1994). SQSTM1/p62 has been shown to promote autophagy by regulating E-cadherin, thereby contributing to poor clinical outcomes (Damiano et al., 2020). Altered cadherin levels can disrupt essential cellular processes, such as water regulation, ion transport, and macromolecule uptake, further exacerbating tumor progression and inflammation (Hu C. A. et al., 2015; Wu et al., 2023).

In this study, elevated ITGAX expression was correlated with greater tumor progression and increased vascular density, suggesting a role in enhancing the efficacy of antiangiogenic therapies. Although our study did not examine the interaction of ITGAX with the tumor microenvironment, this remains an important direction for future research. Further investigation into ITGAX mechanisms using clinical samples will deepen our understanding of its functional role in gastric cancer. In summary, our findings highlight ITGAX as a critical promoter of gastric cancer progression through EMT regulation, with potential links to autophagy and inflammatory responses. ITGAX represents a promising biomarker for early diagnosis, prognosis, and therapeutic targeting in gastric cancer patients. Future studies should aim to explore its broader functional roles and clinical applications to fully elucidate its contributions to cancer biology.

## 5 Conclusion

ITGAX is significantly overexpressed in gastric cancer and strongly correlates with poor clinical prognosis, highlighting its potential clinical relevance. ITGAX promotes cell proliferation, migration, and invasion ability both *in vitro* and *in vivo* by activating the epithelial-mesenchymal transition pathway, characterized by the downregulation of epithelial markers like E-cadherin and upregulation of mesenchymal markers such as N-cadherin and vimentin, facilitates tumor progression and metastasis. These findings suggest that ITGAX may serve as a promising biomarker for early diagnosis, prognosis, and targeted therapy in gastric cancer patients.

## Data Availability

The raw data supporting the conclusions of this article will be made available by the authors, without undue reservation.
